# Enhanced Lithium Recovery from Salt-Lake Brines via Advanced Nanofiltration Membranes: Polymeric Structure–Sieving Performance Relationships

**DOI:** 10.3390/polym17111440

**Published:** 2025-05-22

**Authors:** Ruilin Li, Yong Zheng, Xu Zhang, Mengfei Tan, Jinhui Wang, Guiying Tian

**Affiliations:** 1 Tianjin Key Laboratory of Brine Chemical Engineering and Resource Eco-Utilization, College of Chemical Engineering and Materials Science, Tianjin University of Science and Technology, 13th Avenue 29, TEDA, Tianjin 300457, China; 19922756268@163.com (R.L.); 15038718183@163.com (Y.Z.); zgoodx@mail.tust.edu.cn (X.Z.); m13594894515@163.com (M.T.); 2 Key Laboratory for Special Functional Materials of Ministry of Education, National & Local Joint Engineering Research Center for High-Efficiency Display and Lighting Technology, School of Materials Science and Engineering, and Collaborative Innovation Center of Nano Functional Materials and Applications, Henan University, Minglun Street 85, Kaifeng 475004, China

**Keywords:** salt-lake brine, Li extraction, nanofiltration membrane, positively charged membrane, surface modification

## Abstract

Lithium and its compounds have become crucial energy metals and industrial necessities. Driven by technological advancements and expanding applications in energy storage and portable electronics, ensuring sustainable lithium supply chains is highly important. Thus, the development of efficient extraction methods from salt-lake brines, particularly those with high Mg^2^^+^/Li^+^ ratios, has become a priority. Nanofiltration (NF) separation technology has recently emerged as a key process for selective lithium recovery, presenting remarkable advantages over conventional methods. This review systematically assesses the relationships between the polymeric structure and sieving performance of NF membranes for lithium extraction. This research emphasizes the influence of the membrane architecture on ionic selectivity and permeability. Advanced modification strategies for positively charged NF membranes are meticulously analyzed. These strategies include surface functionalization, copolymer design, and hybrid nanocomposite engineering, all of which are aimed at increasing the Mg^2^^+^/Li^+^ separation efficiency. Moreover, the review delves into innovative membrane module configurations and coupling processes (such as the integration of NF-electrodialysis) to satisfy the requirements of industrial scalability. Finally, the critical challenges and future research directions are highlighted. Our focus lies on cost-effective membrane fabrication, the optimization of long-term stability, and system-level process intensification. This comprehensive analysis not only provides an in-depth mechanistic understanding of high-selectivity lithium extraction from complex brines but also stimulates the rational design of next-generation membranes with precisely tailored ion-transport properties.

## 1. Introduction

With the rapid development of electric vehicles, mobile devices, and stationary energy storage systems, the demand for lithium and its compounds has been increasing rapidly. This trend has not only led to a significant increase in the demand for raw lithium resources but has also propelled the advancement of relevant lithium-extraction technologies. Lithium is relatively abundant in nature, with an average abundance in the Earth’s crust of approximately 0.0065%. In 2023, the global lithium reserves were estimated to be 98 million tons. Lithium exists in two main forms: as lithium oxide (Li_2_O) in lithium-bearing ores, such as spodumene, petalite, lithium-rich clays, and lepidolite, and in ionic form (Li^+^) in lithium-rich brines, including salt-lake brines, underground brines, geothermal waters, and oilfield wastewaters as shown in Table 1 and Table 2. The latter accounts for 66% of the total lithium reserves worldwide [1,2]. When considering the factors related to production cost, the production of one ton of lithium carbonate from self-owned lithium ore incurs a cost ranging from approximately $8000–$11,000. In contrast, the production of one ton of lithium carbonate using lithium-rich brine has a significantly lower cost, ranging from $2800 to $4200. Consequently, the global focus on lithium resource recovery has gradually shifted from lithium ores to lithium-rich brines.

The extraction of lithium from lithium-rich brines involves several challenges. These challenges involve the separation of lithium from brines with low Li^+^ grades and high concentrations of impurity ions (such as Mg^2^^+^ and K^+^), along with the need to increase lithium extraction efficiency and mitigate environmental impacts. In recent years, numerous lithium-extraction technologies have been put into practical application, including chemical precipitation, evaporation crystallization, ion exchange, solvent extraction, and membrane separation. Among these techniques, nanofiltration technology has demonstrated significant potential in the domain of lithium extraction. Owing to its nanoscale pore size and specific surface chemical properties, NF technology is capable of effectively separating Li^+^ ions from interfering ions. This separation mechanism contributes to an increase in the lithium recovery rate and purity. Moreover, through the optimization of the base materials and pore structures of NF membranes, their separation performance and long-term durability can be further improved, consequently reducing the overall cost of the lithium extraction process. Therefore, gaining a deeper understanding and providing a comprehensive summary of recent advancements in nanofiltration technology are highly important.

Currently, membrane-based separation methods for lithium extraction mainly involve electrodialysis and nanofiltration membrane technologies [19]. Electrodialysis represents a membrane separation process in which ion-exchange membranes serve as the medium, and an electrical potential difference acts as the driving force. During the electrodialysis procedure, ions in the solution are impelled to migrate within the membrane under the influence of an electric field. In this process, monovalent-selective ion-exchange membranes are utilized as the separation medium. Monovalent cations, such as Li^+^ ions, can traverse the membrane, whereas divalent cations, such as magnesium ions, are obstructed. This approach effectively differentiates magnesium from lithium, enabling the efficient separation and purification of Li^+^ ions and simultaneously minimizing the coenrichment of other elements [20,21].

NF is a membrane separation technology that lies in the intermediate range between reverse osmosis and ultrafiltration. It functions via pressure-driven mechanisms and is characterized by nanopores with a molecular weight cut-off in the range of 200–1000 Da. NF membranes are capable of retaining divalent and higher-valence ions as well as low-molecular-mass organic substances. However, they display relatively low retention rates for monovalent salts (usually less than 30%). This characteristic renders NF especially suitable for the separation of magnesium and lithium from salt-lake brines. As a pressure-driven membrane separation process, nanofiltration presents distinct advantages in dealing with complex liquid compositions [22].

This review predominantly addresses existing lithium extraction technologies from salt-lake brines, with a specific emphasis on NF membrane technologies. The focal points lie in the preparation and modification of positively charged nanofiltration membranes. These mainly involve the incorporation of functional monomers, as well as modifications using nanomaterials and metal-ion composites. Moreover, this review presents research on the fouling resistance and stability of NF membranes. This review provides further perspectives for enhancing the application of nanofiltration membranes for lithium extraction from salt-lake brines.

## 2. Ionic Screening Mechanisms for NF Membranes

Owing to their operation at relatively low pressures, typically in the range of 0.3–3.0 MPa, NF membranes are often denoted as low-pressure reverse osmosis [23,24]. NF membranes are characterized by nanopores with sizes ranging from approximately 0.5–2.0 nm and predominantly retain substances with molecular weights in the interval of 200–2000 Da [25,26]. These membranes generally carry an electrostatic charge. This property endows them with the ability to efficiently remove suspended particles, colloids, bacteria, and organic molecules. Simultaneously, they retain substances bearing the same charge and allow the passage of substances with opposite charges. Moreover, nanofiltration enables the selective separation of mono- and multivalent ions. The electrostatic effect becomes more pronounced as the charge increases, which is particularly significant for the separation of lithium and magnesium ions in salt-lake brines [27,28].

**Figure 1:**
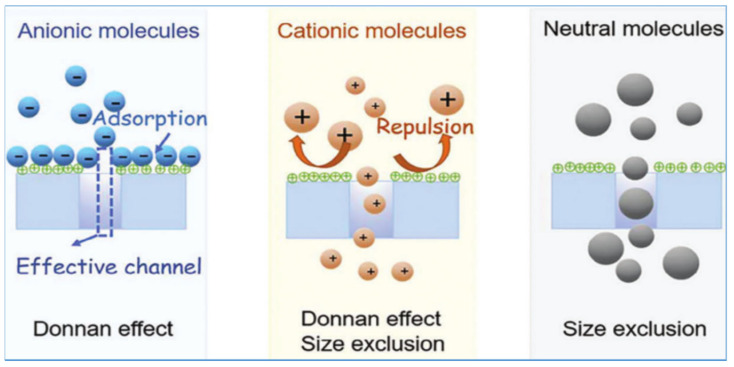
Schematic diagram of size-sieving effect and Donnan effect in NF membranes [29].

NF membranes are pressure-driven separation membranes with complex mass-transfer mechanisms. With respect to the structure and functionality of NF membranes, the sieving effect exerted by the pore size on the membrane surface represents one of the crucial influencing factors. Subsequent to interfacial polymerization, the membrane surface is subjected to electrostatic effects, giving rise to a certain number of charged groups. These charged groups display diverse electrical properties (positive, negative, or neutral) within specific reaction environments. As a result, they generate either attractive or repulsive forces towards the ions in the liquid phase, thereby influencing the separation efficiency. Furthermore, the transport process within pores is also affected by the dielectric properties inherent to the membrane pores. In general, the separation mechanism of NF membranes principally encompasses three aspects: nanopore sieving, Donnan electrostatic effects, and dielectric repulsion within the membrane pores [29] (see Figure 1).

### 2.1. Nanopore Sieving Effect

Pore sieving operates based on the size of the membrane pores. It selectively permits smaller ions to permeate while intercepting larger ions. During the fabrication of NF membranes, a separation layer is predominantly formed via interfacial polymerization. In this process, a diverse range of pore sizes (ranging from 0.5 to 2.0 nm) are generated through cross-linked structures. This enables Li^+^ ions to pass through relatively easily, while having a certain interception effect on Mg^2+^ ions, thereby facilitating mass transfer and selective separation [30]. This mechanism proves highly efficient for larger, electrically neutral solutes. However, it fails to completely account for the separation mechanism of charged ions and solutes.

In the actual Li^+^/Mg^2+^ separation process, a combination of multiple membranes is often used. For instance, ultrafiltration membranes are first employed for pretreatment, followed by the preliminary separation of Li^+^ and Mg^2+^ ions through nanofiltration membranes. Finally, reverse osmosis membranes are used for the concentration and purification of Li^+^ ions. This combined membrane process can fully utilize the pore size sieving characteristics of different membranes to perform the hierarchical separation of ions and impurities with different particle sizes.

### 2.2. Donnan Electrostatic Effect

The charged characteristics of NF membranes enable them to either attract or repel charged ions in the solution, thereby exerting an influence on ion transport. Through the incorporation of ionizable side groups, the surfaces of NF membranes can be rendered positively or negatively charged. As a result, ions with different charges are attracted or repelled, thereby achieving separation. Typically, positively charged membrane surfaces bear alkaline groups such as primary, secondary, tertiary, and quaternary ammonium groups. Conversely, negatively charged surfaces generally carry acidic groups such as carboxylic, sulfonic, and hydroxyl groups [31].

When the solute ions possess an opposite charge to that of the membrane, they are electrostatically attracted into the pores. Conversely, if they carry the same charge as the membrane, they are repelled by electrostatic forces. The phenomenon whereby the presence of fixed charge groups on the membrane surface leads to the formation of an asymmetric distribution of mobile ions across the membrane and generates a transmembrane potential difference is termed the Donnan effect [32]. The core of this effect lies in the fact that ions with the same charge as the membrane are electrostatically repelled and enriched in the solution phase, while counterions with opposite charges undergo cooperative migration under electroneutrality constraints. This process ultimately establishes a thermodynamic equilibrium state (i.e., Donnan equilibrium) across the membrane [33], driven by charge balance. The resulting transmembrane electric potential (Donnan potential) directly influences the diffusion and transport rates of ions [34,35]. Therefore, the charge properties and charge intensity of the NF membrane substantially affect its separation efficiency. The ion-separation performance of NF membranes is associated not only with the Donnan effect but also with the nanopore sieving process.

In practical NF separation processes, NF membranes display a relatively low retention rate for monovalent ions such as Li^+^, Na^+^, and K^+^. However, they exhibit a high retention efficiency for multivalent ions such as Mg^2^^+^ and SO_4_^2−^ [25]. Furthermore, electronegative NF membranes demonstrate excellent retention capabilities for divalent and higher-valence anions, including SO_4_^2−^, PO_4_^3−^, and SO_3_^2−^. In contrast, positively charged NF membranes show enhanced retention of high-valence cations [36]. Leveraging these selective properties, positively charged nanofiltration technology can be utilized to efficiently separate lithium/magnesium ions from salt-lake brines.

### 2.3. Dielectric Repulsion in Membrane Pores

The dielectric effect is another key mechanism for ion-selective separation in nanofiltration membranes. The polarization caused by hydrated ions within membrane pores leads to a decrease in the membrane’s dielectric constant [37]. When the dielectric constant in the membrane pores (ε_p_) is lower than that of the bulk solution (ε_β_), two effects occur: first, the ion solvation energy barrier (Born effect), where ions must overcome a solvation energy barrier when entering the membrane pores; this barrier affects the distribution of ions inside and outside the pores, with the distribution being proportional to the square of the ion valence and inversely proportional to the ion radius, leading to preferential retention of high-valence ions. Second, the image force effect, in which dielectric differences between the membrane and water induce interfacial polarization, generating opposite charges that hinder ion diffusion [38]. As more counter-charged ions continuously enter the pores, the repulsive effect intensifies, promoting the secondary separation and retention of coexisting ions [23,39].

The dielectric effect is less influenced by solution ionic strength and pH, exhibits better stability than the Donnan effect in high-salt environments, and works in conjunction with steric hindrance to enable efficient retention of multivalent ions (e.g., Mg^2^^+^, SO_4_^2−^). This makes it particularly suitable for applications such as magnesium–lithium separation in salt-lake brines, offering both operational performance advantages and cost-effectiveness [40].

During the transport process, the membrane pore confinement effect further reduces the dielectric constant of water, thereby enhancing the energy barrier effect. Studies have found that when the membrane pore size is between 0.35 and 0.44 nm (e.g., 0.34 nm for the NF90 membrane), the dielectric effect collaborates with steric hindrance: monovalent ions (e.g., Li^+^, hydrated radius 0.340 nm) preferentially permeate due to their lower energy barrier, while multivalent ions (e.g., Mg^2^^+^, hydrated radius 0.428 nm) are retained [38]. In high-salt environments, increased ionic strength weakens the Donnan effect, making the dielectric effect the dominant mechanism [41]. However, due to the elusive and non-intuitive nature of the dielectric effect, and extensive research indicating that the strength of dielectric repulsion is influenced by multiple factors such as pore geometry, membrane material, and solution properties, its accurate quantification remains challenging. Current studies often rely on semi-empirical models and oversimplify the system, rendering research on this phenomenon an interesting yet challenging task in the academic community.

## 3. Research Progress on Li Extraction Using Commercial Nanofiltration Membranes

NF membrane technology was introduced in research on NS-300 by Filtec in the 1970s. Through successive rounds of research and development, it gradually evolved and entered commercial production. At present, among the most representative membrane materials are Dow Filmtec’s NF270 and NF90 membranes. These membranes are renowned for their outstanding desalination performance and high-flux capabilities. Moreover, GE Water & Process Technologies manufactures the Osmonics-HL8040F 400 and DK series of NF membranes, which have also found applications in the research of lithium extraction from salt-lake brines.

**Figure 2:**
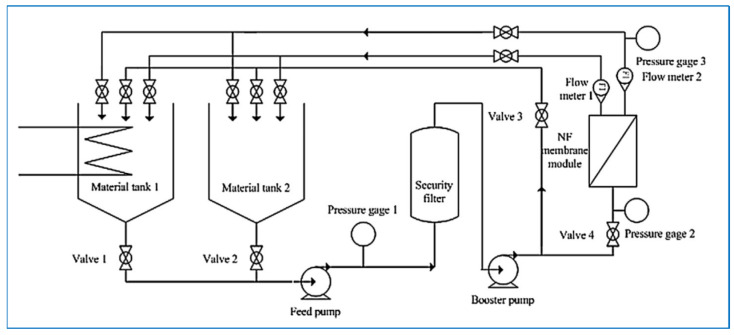
Experimental setup for testing the separation efficiency of the DL-2540 NF membrane [44].

In Western China, the majority of salt-lake brines feature high magnesium-to-lithium ratios. Moreover, given the comparable solvation properties of magnesium and lithium ions, the extraction of lithium with high selectivity presents a formidable challenge. Yang et al. [42] undertook lithium-extraction research using DKNF membranes in mixed solutions containing Mg^2^^+^, Li^+^, and Cl^−^. Their results revealed that the separation of Mg^2^^+^ from Li^+^ is chiefly governed by the operating pressure (or permeate flux), whereas the Mg^2^^+^/Li^+^ ratio and the initial lithium concentration have relatively little influence on the separation process. Additionally, they probed the impact of the effective membrane charge density on the selective separation of magnesium and Li^+^ ions. Through their efforts, they managed to significantly reduce the magnesium-to-lithium ratio in the brine from 18–24 to approximately 6, with a maximum separation factor of approximately 0.31. This achievement effectively demonstrated the preeminent performance of the DK NF membranes. Bi et al. [43] reported that Donnan electrostatic repulsion, dielectric repulsion, and spatial steric hindrance all have negative impacts on mass transfer within NF membranes. Their experimental results indicated that NF technology is capable of effectively recovering Li^+^ from brines with high Mg^2^^+^/Li^+^ ratios. When the operating pressure reached 0.8 MPa and the Mg^2^^+^/Li^+^ ratio was 40, the magnesium retention rate (R_Mg_^2^^+^) and separation factor (SF) were 0.96 and 42, respectively. The Mg^2^^+^/Li^+^ ratio in the permeate decreased to 0.9, and the recovery rate of Li^+^ was 85%. Sun et al. [44] explored the separation efficiency of the DL-2540 NF membrane, taking Dongtaijier brine as an example as shown in Figure 2. They discovered that the extent of magnesium-lithium separation is highly reliant on the Mg^2^^+^/Li^+^ ratio, operating pressure, and pH value. When the Mg^2^^+^/Li^+^ ratio was less than 20, these three factors significantly influenced the separation factor (SF). Conversely, when the ratio exceeded 20, the SF remained nearly unchanged. Moreover, under constant conditions, increasing the pressure, decreasing the pH, and reducing the temperature (albeit with a limited impact) were operational parameters more conducive to lithium extraction. Additionally, through comprehensive data simulation, they deduced the relationship between the rejection rates of Mg^2^^+^ and Li^+^. This finding offers valuable insights for optimizing NF membranes in the separation of lithium and magnesium from brines with high Mg^2^^+^/Li^+^ ratios.

Somrani et al. [45] employed the NF90 nanofiltration membrane to extract lithium from diluted brines sourced from Tunisian salt lakes, which were diluted by a factor of ten. Research has demonstrated that under low-pressure conditions (<15 bar), the separation process is highly efficient, attaining a 100% magnesium retention rate and a 15% lithium retention rate. This study further explored the underlying reasons for this high separation efficiency. These reasons included a higher hydraulic permeability to pure water (L_p_ = 15 L h^−1^ m^−2^ bar^−1^) and a relatively high hydraulic permeability to a 0.1 M NaCl solution (L_p_ = 7.5 L h^−1^ m^−2^ bar^−1^). Additionally, at lower operating pressures (below 15 bar), there was an approximately 40% increase in the selectivity for monovalent ions. The study also compared the NF90 membrane and the low-pressure reverse osmosis XLE membrane. The results indicated that at 20 °C, the NF90 membrane achieved complete separation between lithium and sodium in a 0.1 M NaCl solution, with a diffusion flux of 4.42 × 10^−7^ mol s^−1^ m^−2^, which was five times greater than that of the XLE membrane.

## 4. Application of Positively Charged NF Membranes in Li Extraction from Salt-Lake Brines

Owing to their positive surface charge, positively charged NF membranes exploit the electrostatic repulsion effect. In this context, the membrane surface exerts substantially stronger repulsive forces against divalent cations (Mg^2^^+^) than against monovalent cations (Li^+^). As a result, Li^+^ ions can more easily traverse the membrane, while divalent magnesium ions are retained, thereby enabling the efficient separation of lithium and magnesium ions [46]. In recent years, researchers have been attempting to develop high-performance positively charged NF membranes specifically tailored for lithium—magnesium separation [47]. Currently, the fabrication of positively charged NF membranes involves a diverse range of methods and base materials. Key technological strategies involve interfacial polymerization and surface modification techniques. The main materials utilized are chitosan (CS), polyethersulfone (PES), polyethyleneimine (PEI), and cellulose acetate (CA). The multiplicity of preparation methods and the selection of various materials for positively charged membranes provide innovative approaches and effective results for the application of NF membranes in lithium extraction from salt-lake brines.

### 4.1. Positively Charged Modification of the Amine Group-Based Monolayer NF Membrane

**Figure 3:**
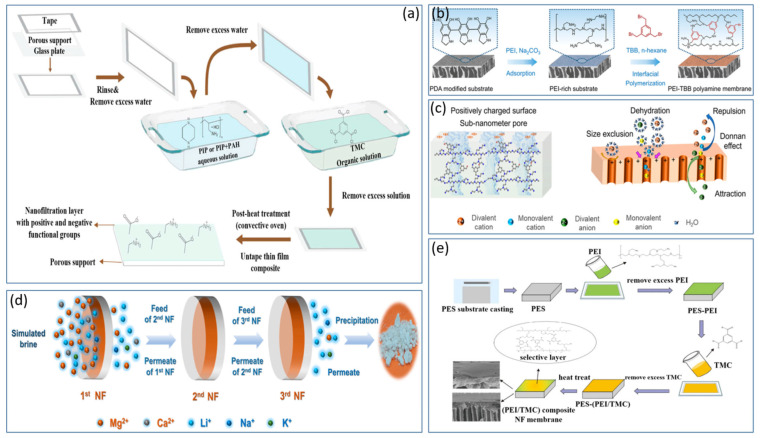
Synthesis route for PIP/PAH-PS35 membrane using interfacial polymerization (IP) method (**a**) [48]; schematic diagram for the fabrication of PEI–TBB composite membrane via interfacial polymerization (**b**) [49]; schematic of PEI–TBB composite membrane surface featuring positively charged and sub-nanometer pores and the ion separation mechanism for different valence states (**c**) [49] and three-stage NF process schematic for treating simulated salt-lake brine (**d**) [49] (the composition of the simulated brine used in the manufacturing process of the composite NF membrane is as follows: 5940 mg·L^−1^ MgCl_2_, 182 mg·L^–1^ LiCl, 763 mg·L^−1^ NaCl, 573 mg·L^−1^ KCl and 83 mg·L^–1^ CaCl_2_ (**e**) [50]).

The morphology of the separation layer in NF membranes is a crucial factor determining their separation performance. The type of amine monomer not only impacts the morphology of the separation layer but also affects the rate of interfacial reactions. At present, the preparation of positively charged NF membranes via amine-group modification is garnering increasing favor within the academic community. Dey et al. [47] employed polyethyleneimine (PEI) to react with glycidyl trimethylammonium chloride (GTACl) to generate quaternary ammonium salts, which were subsequently anchored onto a composite positively charged NF membrane. Through in situ interfacial polymerization, they fabricated positively charged thin-film composite (TFC) membranes using functionalized PEI and terephthaloyl chloride. The findings demonstrated that higher curing temperatures (within a temperature gradient of 110–125 °C) and longer curing times (within a time gradient of 10–30 min) could augment the surface density of the membrane. Consequently, this led to a reduction in the solvent flux and an increase in the solute retention rates. The retention order of the corresponding salts was as follows: CaCl_2_ > Na_2_SO_4_ > NaCl > MgSO_4_. Pizarro et al. [48] employed interfacial polymerization (IP) to introduce additional weakly basic functional groups into positively charged NF membranes (PIP/PAH-PS35) by adding polyallylamine hydrochloride (PAH) as depicted in Figure 3a. These membranes demonstrated excellent removal rates for rare-earth elements, Ca^2^^+^, and Mg^2^^+^ under conditions of high ionic strength, low pH, low permeate flux, and high water recovery. At an ionic strength as high as 100 mM, the retention rate of Na^+^ reached 99.3%, highlighting the remarkable selective separation potential of positively charged NF membranes under low-permeate-flux conditions. Zhao et al. [49] carried out interfacial polymerization on a substrate modified with polydopamine (PDA) using polyethyleneimine (PEI) and tribromobenzene (TBB) as shown in Figure 3b. In Figure 3c, this process led to the formation of an amine-linked PEI–TBB selective layer with an ultrathin thickness of 95 nm. At a pH value of 7, the zeta potential of this layer was +20.9 mV, thereby resulting in a composite positively charged NF membrane. The PEI–TBB composite membrane exhibited a permeability of 4.2 L·m^−2^·h^−1^·bar^−1^ for the NaCl/MgCl_2_ and LiCl/MgCl_2_ mixtures. The rejection rates exceeded 90% for various divalent salts and separation factors greater than 15. Further investigations revealed that the use of a triple-stage NF process rapidly decreased the Mg^2^^+^/Li^+^ ratio from 50 to 0.11, with an overall separation factor (SF) of 455 as depicted in Figure 3d. This effectively enabled the separation of lithium and magnesium in salt-lake brines. In Figure 3e, Xu et al. [50] utilized a polyethersulfone ultrafiltration membrane as a substrate and performed interfacial polymerization with polyethyleneimine (PEI) and trimesoyl chloride (TMC) to design and construct a composite positively charged NF membrane abundant in −NH_3_^+^ and −NH_2_^+^ groups. Zeta potential measurements revealed that at pH values below 9.3, the membrane exhibited a substantial positive charge attributable to the numerous unreacted amine groups on the PEI branches. Under an operating pressure of 8 bar, the composite positively charged NF membrane was employed to filter salt-lake brine with a high lithium-to-magnesium ratio. The results demonstrated that its pure-water permeability was 5.02 L h^−1^ m^−2^ bar^−1^. Moreover, the Mg^2^^+^/Li^+^ ratio decreased from an initial value of 20 to 1.3. The disparity in removal rates between Mg^2^^+^ and Li^+^ reached as high as 76%. Compared with that of conventional membranes, the separation performance of this composite NF membrane more than doubled. The study further reported the retention and desalination rates of the composite NF membrane for specific salts, as detailed below: MgCl_2_ (94.8%) > MgSO_4_ (84.1%) > Na_2_SO_4_ (81.4%) > NaCl (36.9%) > LiCl (30.6%).

**Figure 4:**
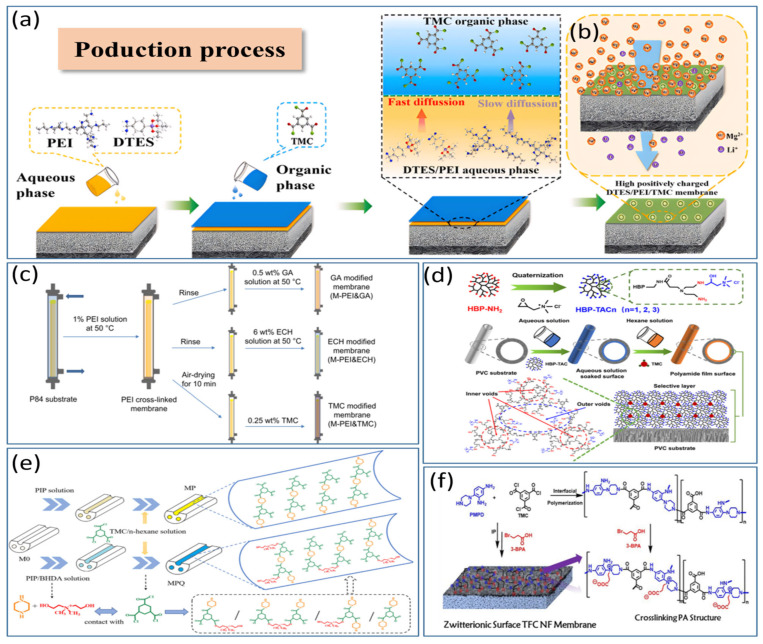
Preparation process of DTES/PEI/TMC TFN membrane for lithium separation from simulated brine (**a**) [51]; membrane separation performance in simulated brine (**b**) [51]; modification processes of three types of membranes (**c**) [55]; NF membrane fabrication process via internal surface IP (**d**) [57]; synthetic process of HBP-TAC_n_ (n = 1,2,3) and the preparation of NF membranes with ultrathin selective layers and chemical structure schematics (**e**) [58]; schematic of high-performance TFCNF membrane preparation using zwitterionic surface modification IP method with novel monomers PMPD in water phase and TMC in oil phase (**f**) [59].

Wu et al. [51] mixed polyethyleneimine (PEI) with a monoamine silane coupling agent (3-diaminomethyl-cyclohexyl triethoxysilane, DTES) and employed interfacial polymerization with trimesoyl chloride (TMC) to synthesize a novel positively charged thin-film nanocomposite NF membrane (DTES/PEI/TMC TFN) as shown in Figure 4a. This study further involved SiO_2_ doping to increase the surface hydrophilicity and positive charge. The results indicated that the water flux of the DTES/PEI/TMC membrane was approximately 1.4 times higher than that of the PEI/TMC membrane. Moreover, the composite membrane exhibited retention rates of 91.46% for Mg^2^^+^ and 10.5% for Li^+^, with a separation factor (SF) of 12.95 as depicted in Figure 4b. These performance metrics surpassed those of commercial membranes, rendering them suitable for lithium-extraction processes from salt-lake brines with high lithium-to-magnesium ratios. Zhe et al. [52] carried out interfacial polymerization on a polydopamine-coated hydrolyzed polyacrylonitrile substrate, employing polyethyleneimine (PEI) and trimesoyl chloride (TMC) to synthesize a positively charged NF membrane. The retention rate of divalent ions (Mg^2^^+^) was substantially higher than that of monovalent ions (Na^+^, Li^+^). Specifically, the retention rates were 93.6 ± 2.6% for MgSO_4_, 92.4 ± 1.3% for MgCl_2_, and 90.4 ± 2.1% for Na_2_SO_4_. The study further revealed that under an operating pressure of 8 bar, the permeate flux was 17.2 ± 2.8 L∙m^−2^∙h^−1^, and the retention rate for NaCl was 27.8 ± 2.5%. Yang et al. [53] successfully synthesized a polyamide NF membrane via an emulsion-mediated interfacial polymerization approach. In this process, a hydrophobic and oleophilic polytetrafluoroethylene (PTFE) membrane was employed to adsorb, coalesce, and transport ethane droplets containing trimesoyl chloride (TMC) molecules from an emulsion. These droplets then reacted with piperazine (PIP) at the interface. By investigating the surface morphology, thickness, and chemical properties of polyamide membranes at different time intervals, this study systematically elucidated the controlling effects of interfacial polymerization. Compared with traditional interfacial polymerization methods, the results demonstrated that the polyamide layer formed on the PTFE membrane was thinner and smoother. This membrane exhibited a water permeability of 7.3 L h^−1^ m^−2^ bar^−1^ and a Na_2_SO_4_ retention rate of 92.3%. Bi et al. [54] selected piperazine (PIP) and trimesoyl chloride (TMC) for interfacial polymerization to synthesize a positively charged NF membrane and incorporated zwitterionic-functionalized carbon nitride (BHC-CN). When simulated brine with a lithium-to-magnesium ratio of 73 was filtered, the results indicated that the lithium-to-magnesium ratio of the filtered brine decreased to 1.85. This demonstrated the outstanding performance of the membrane and provided a novel approach for lithium extraction from salt-lake brines with high lithium-to-magnesium ratios.

In Figure 4c, Gao et al. [55] modified a base membrane with glutaraldehyde (GA), epichlorohydrin (ECH), and P84 polyimide, which is a copolymer of 3,3′,4,4′-biphenyltetracarboxylic dianhydride with 80% toluene diisocyanate and 20% methylene diphenyl diisocyanate (BTDA-TDI/MDI). They used these materials to create an outer-selective thin-film composite (TFC) hollow-fiber NF membrane. This synthesis process does not require aliphatic solvents, making it environmentally friendly, and the three modified materials enhance the salt resistance of the membrane in simulated brine. The membrane shows high rejection of salts such as Na_2_SO_4_, NaCl, and MgSO_4_. The introduction of more amino groups by P84 led to improved performance of the membrane. The retention sequence for the PEI-GA composite membrane was MgSO_4_ > MgCl_2_ > NaCl > Na_2_SO_4_, with retention rates all exceeding 90%. The pure water permeability of the membrane was 1.747 ± 0.01 L h^−1^ m^−2^ bar^−1^, indicating good stability.

Gu et al. [56] modified polyethyleneimine (PEI) membranes by incorporating melamine, resulting in a PEI/melamine NF membrane that demonstrated increased permeate flux for simulated saline solutions, high selectivity, and excellent desalination performance. The modified membrane showed a retention rate for MgCl_2_ of over 90%. Mu et al. [57] used a PES (polyethersulfone) tri-channel capillary ultrafiltration (UF) membrane as the substrate to introduce a mixture of bis(2-hydroxyethyl)dimethyl ammonium chloride (BHDA) and piperazine (PIP) to create a new type of polyamide NF membrane as shown in Figure 4d. The results indicated that the newly fabricated PIP/BHDA-TMC (MPQ) membrane achieved a pure water flux of 12.9 L h^−1^ m^−2^ bar^−1^, which is 2.4 times greater than that of the original membrane. The desalination sequence and corresponding retention rates were as follows: MgSO_4_ (93.46%) > Na_2_SO_4_ (91.61%) > MgCl_2_ (88.15%) > NaCl (22.03%), with heavy metal retention rates above 84%. Qiu et al. [58] utilized cationic hyperbranched polyamines (HBPs) and trimesoyl chloride (TMC) for interfacial polymerization on the surface of polyvinyl chloride ultrafiltration (PVC-UF) hollow-fiber membranes as depicted in Figure 4e. By introducing varying numbers of quaternary ammonium groups on the surface of cationic HBP molecules, they were able to regulate the size and charge properties of the outer voids, achieving high permeability and molecular selectivity. The resulting NF membrane exhibited an average water flux of 310.5 L h^−1^ m^−2^ bar^−1^ and a MgCl_2_ retention rate of 92.0%. In Figure 4f, Li et al. [59] designed a novel amine monomer, 4-(piperazin-1-yl)benzene-1,3-diamine (PMPD), by directly attaching PIP moieties to the phenyl rings of m-phenylenediamine (MPD). This monomer was used with TMC to fabricate an NF membrane, which was then modified with 3-bromopropionic acid (3-BPA). The modified PMPD-PA membrane had a pure water permeability of 19 L h^−1^ m^−2^ bar^−1^ and retention rates of 98.4% for Na_2_SO_4_ and 24.1% for NaCl. Zheng et al. [60] used polypropylene hollow-fiber microfiltration membranes as substrates and coated them with a layer of polyvinyl alcohol and a polyquaternary ammonium-10 (a reaction product of hydroxyethyl cellulose with trimethyl substituted epoxides, PQ-10) via a dip-coating method. The coated surface was then crosslinked with glutaraldehyde under acidic conditions to prepare hollow-fiber NF membranes. The study revealed that these membranes remained positively charged across a pH range of 3.0–9.0. Under neutral pH conditions, the retention sequence for simulated brine was CaCl_2_ > MgCl_2_ > NaCl > MgSO_4_ > Na_2_SO_4_, with a pure water flux of approximately 25.7 L h^−1^ m^−2^ bar^−1^. When a 500 mg/L electrolyte solution was filtered at an operating pressure of 3 bar, the retention rates for CaCl_2_ and NaCl were 92.8% and 35.0%, respectively.

### 4.2. Modification of NF Membranes with Positively Charged Intermediate Composite Layers

**Figure 5:**
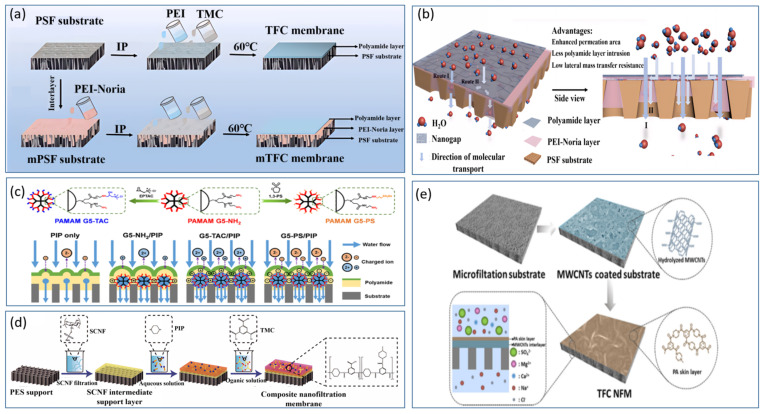
Manufacturing and diffusion principles of positively charged NF membrane (**a**) [61]; dynamic water contact angle (**b**) [61]; synthesis process of ionic PAMAM dendrimers and the screening/transport mechanisms of mono/divalent co-ions through PA membranes (**c**) [62] (the polyamide membranes without PAMAM dendrimers and those containing PAMAM G5-NH2, PAMAM G5-TAC, and PAMAM C5-PS are denoted as PIP only, G5-NH2/PIP, G5-TAC/PIP, and G5-PS/PIP, respectively); schematic of CNF-TFC NF membrane fabrication (**d**) [64]; schematic of composite membrane combining carbon nanotube intermediate layer and microfiltration support with high NF performance (**e**) [66].

The effective use of intermediate composite layers in high-performance positively charged NF membranes can create a unique nanocomposite morphology, significantly enhancing the separation capabilities of NF membranes. Wang et al. [61] developed an interlayer-mediated positively charged NF membrane, introducing a novel method for fabricating high-performance NF membranes via interlayer technology. Using polyethyleneimine (PEI) and the macrocyclic polyphenol molecule Noria as raw materials, they employed interfacial polymerization and a rapid codeposition surface modification technique to fabricate a composite NF membrane as shown in Figure 5a. The results revealed that unique nanocrystals of Zeolitic Imidazolate Framework-8 (ZIF-8) were synthesized on the surface of the Noria-PEI-modified intermediate deposition layer, enhancing the transverse mass transfer process of the modified composite membrane. In Figure 5b, it exhibited a high water permeability of 110.4 L h^−1^ m^−2^ MPa^−1^, which is 2.6 times greater than that of the original membrane, with a MgCl_2_ retention rate of up to 95.4%. Qiu et al. [62] synthesized ion-type PAMAM dendrimers with terminal quaternary ammonium groups via poly(amidoamine)-amine (PAMAMG5-NH_2_) dendrimers and epichlorohydrin trimethyl ammonium chloride (EPTAC) through interfacial polymerization. They integrated these dendrimers into polyamide (PA) membranes to form nanochannels. Using a mixture of PAMAMG5-TAC and PIP as the aqueous phase solution and TMC as the organic solvent, they prepared a positively charged NF membrane as depicted in Figure 5c. The results indicated that the membrane achieved a divalent Mg^2+^ retention rate of 97.0%, with a pure water permeability of 315 L h^−1^ m^−2^ MPa^−1^, demonstrating exceptional performance in terms of selective ion separation and high permeability. Zhu et al. [63] utilized a polyacrylonitrile ultrafiltration membrane as a base, with piperazine (PIP) and trimesoyl chloride (TMC) as monomers, to fabricate a high-performance MG-NF membrane through interfacial polymerization. They incorporated quaternized cross-linked microgels (PNI6) as an intermediate layer. The introduction of PNI6 improved the hydrophilicity and increased the positive charge on the membrane surface. At 25 °C, the MG-NF-6 membrane with PNI6 achieved a water flux of 10.1 L h^−1^ m^−2^ bar^−1^, with a MgCl_2_ retention rate of 93.4%. The results indicated that as the water temperature increased, the water flux of the MG-NF-6 membrane significantly improved, increasing by 1.3 times at 45 °C, although the MgCl_2_ retention rate decreased to 90%. Li et al. [64] used a polyethersulfone (PES) ultrafiltration membrane as a base and cellulose sulfate nanofibers (SCNFs) as an intermediate layer. They then conducted interfacial polymerization with PIP and TMC on the surface to create a three-layer high-performance NF membrane as shown in Figure 5d. They reported that the modified membrane had a water flux of 30 h^−1^ m^−2^, and the salt retention sequence rates were as follows: Na_2_SO_4_ (98.91%) > MgSO_4_ (78.42%) > MgCI_2_ (49.80%) > NaCI (21.23%) > CaCI_2_ (12.31%). Li et al. [65] introduced layered double hydroxides (LDHs) as intermediate layers and used TMC and PIP as monomers for interfacial polymerization to fabricate a polyamide membrane. This study also explored the differences in membrane thickness and performance with different concentrations of PIP. The results showed that NF membranes prepared with higher concentrations of PIP exhibited excellent divalent salt retention capabilities, with removal rates for MgCl_2_ and Na_2_SO_4_ of 95.1% and 97.1%, respectively, although the pure water flux was lower than that of membranes made with lower concentrations of PIP, reaching only 28 L h^−1^ m^−2^ bar^−1^. Wu et al. [66] introduced multiwalled carbon nanotubes (MWCNTs) as an intermediate layer deposited on a microfiltration membrane, followed by interfacial polymerization to produce a composite NF membrane. In Figure 5e, the introduction of the intermediate layer stores amino groups in the interlayers of the MWCNTs to regulate interfacial polymerization. This composite NF membrane had a water flux that was twice that of the original membrane, reaching 105.4 L h^−1^ m^−2^, with a Na_2_SO_4_ retention rate of 95%. Ma et al. [67] used zwitterionic grafted carbon nitride (g-C3N4) nanosheets as an intermediate layer coprecipitated with polydopamine (PDA) to prepare a positively charged NF membrane. The results indicated that the membrane-treated simulated brine (MLR of 84) had a Mg^2+^ retention rate of 96.66%, and a Mg^2+^ retention rate of 97.90% was achieved when actual brine was treated.

### 4.3. Modification of NF Membranes with Composite Inorganic Nanomaterials

**Figure 6:**
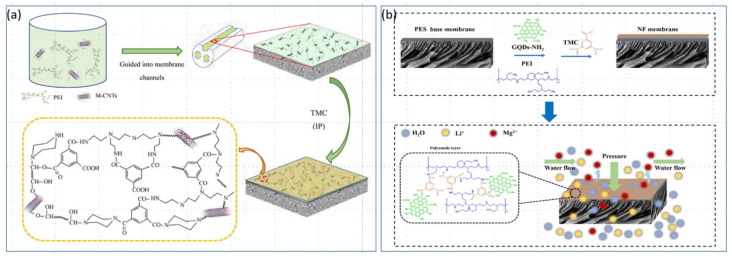
Modified NF membrane fabrication process (**a**) [68]; manufacturing and filtration process scheme for amine-functionalized graphene quantum dot-doped modified membrane (**b**) [70].

In recent years, new composite materials have been increasingly discussed. Nanomaterials inherently possess nanopores. By incorporating a certain amount of nanomaterial, it is possible to increase the porosity of NF membranes and improve the separation efficiency of Mg^2+^/Li^+^. Zhang et al. [68] used a polyethersulfone (PES) trichannel capillary ultrafiltration membrane as a substrate and polyethyleneimine (PEI) as a precursor to prepare a positively charged NF membrane through interfacial polymerization. This study involved adding 0.01 wt.% multiwalled carbon nanotubes (MWCNTs) to an aqueous solution. The hydroxyl-modified MWCNTs (MWCNTs-OH) were grafted with piperazine (PIP) and incorporated into the intermediate selective layer to create a modified composite positively charged NF membrane as shown in Figure 6a. The results revealed that at an operating pressure of 4 bar, the water flux of the modified NF membrane increased nearly 2.7-fold. Additionally, the retention rates for divalent cations such as Mg^2+^ and Ca^2+^ in the modified composite positively charged NF membrane increased to over 97%, whereas the retention rates for the monovalent cations Na^+^ and Li^+^ were lower (<70%). The modified NF membrane also exhibited good separation efficiency and durability for Mg^2+^ and Li^+^ in a mixed salt solution mimicking salt-lake brine composition. This research contributes to technological innovations in recovering lithium from salt-lake brines with high Mg^2+^/Li^+^ ratios. In the research by Ma et al. [69], two types of metal—organic frameworks (MOFs), NH_2_-MIL-101(Al) and NH_2_-MIL-101(Cr) (MIL = Institute Lavoisier Materials), were incorporated into a chitosan polymer matrix to prepare new positively charged NF membranes. Their study explored the impact of MOF morphology on NF performance and revealed that at the same concentration of MOFs, the Al-based membrane exhibited twice the water flux as the Cr-based membrane did, although both materials presented similar retention rates of approximately 93%, with the retention sequence being MgCl_2_ > CaCl_2_ > NaCl > Na_2_SO_4_. The results demonstrated the potential of MOFs to increase the ionic selectivity of NF membranes. Xu et al. [70] incorporated amine-functionalized graphene quantum dots (GQDs-NH_2_) into the aqueous phase (PEI) to prepare a new type of NF membrane as depicted in Figure 6b. The optimal concentration of GQDs-NH_2_ was found to be 0.03 wt.%, at which the NF-0.03 membrane had a high positive charge. The separation SF factor for simulated brine was lower, but the separation efficiency for magnesium and Li^+^ ions was excellent, with retention rates of 97.16% for Mg^2+^ and 20.02% for Li^+^. This adjustment lowered the Mg^2+^/Li^+^ ratio in the solution from 20 to 0.7. The permeate flux of the NF-0.03 membrane was nearly 1.4 times greater than that of the original NF membrane, demonstrating the feasibility of using GQDs-NH_2_ to improve the performance of NF membranes for Li extraction.

**Figure 7:**
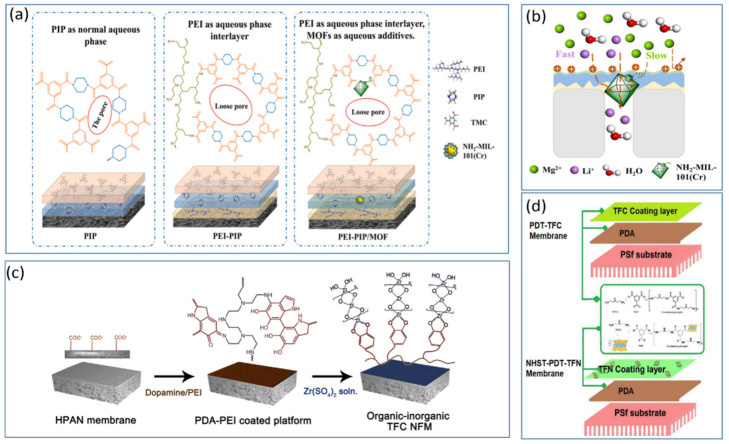
Schematic of PEI-PIP/MOF TFN membrane fabrication (**a**) [71]; diagram of Li^+^ and Mg^2+^ transport through MOF channels (**b**) [71]; schematic of organic–inorganic TFC NFM fabrication and mechanism using ultra-thin ZrO_2_ film as a selective layer on PDA-PEI coated polyacrylonitrile membrane (**c**) [72]; manufacturing process flow for PDT-TFC and NHST-PDT-TFN membranes (**d**) [73].

Ion selectivity is a crucial indicator of the performance of NF membranes. Modifying NF membranes with doped metal particles and metal nanomaterials can further enhance the ion-selective separation capabilities of the membranes and promote the enhancement of positive charges on their surfaces. Additionally, some doped metal particles can also contribute to the antimicrobial and anti-fouling properties of NF membranes. Jia et al. [71] utilized NH_2_-MIL-101(Cr) nanoparticles (NPs) to modify NF membranes, incorporating these NPs into a polyamide layer situated atop a PEI layer to create a positively charged composite PEI-PIP/MOF TFN membrane as shown in Figure 7a. In Figure 7b, the study indicated that NH_2_-MIL-101 (Cr) provides an efficient mass transfer pathway for Li^+^ and, owing to the introduction of -NH_2_ groups, enhances the positive charge, facilitating the separation of lithium and magnesium. The results demonstrated that the membrane achieved a retention rate of 42.3% for LiCl and 96.1% for MgCl_2_, with a pure water permeability of 17.0 L h^−1^ m^−2^ bar^−1^. The selectivity for S (Li, Mg) was 102, and the membrane displayed good stability. Lv et al. [72] codeposited polydopamine (PDA) and PEI on a polyacrylonitrile (HPAN) ultrafiltration membrane and modified it with zirconium oxide (ZrO_2_) nanoparticles to fabricate an organic—inorganic TFCNF membrane (TFC NFM). The resulting zirconium oxide selective layer improved the selectivity for divalent cations as presentedin Figure 7c. The study revealed that the organic—inorganic TFC NFMs retained more than 90% of the divalent cations, such as Mg^2+^, with a membrane flux of 60 L h^−1^ m^−2^ at pH 6 and an operating pressure of 0.6 MPa. The salt retention sequence was MgCl_2_ > CaCl_2_ > MgSO_4_ > NaCl > Na_2_SO_4_, and the membrane operated stably for extended periods. Karki et al. [73] synthesized a new type of TFN membrane, HHST-PDT-TFN, by using diethylenetriamine (DETA) and TMC monomers to synthesize talc powder (NHST) nanosheets on a polydopamine (PDA) coating as depicted in Figure 7d. They explored the impact of different concentrations of NHST nanosheets on membrane performance. The results showed that a 0.5% NHST-incorporated HHST-PDT-TFN2 membrane had retention rates of 98.96% for Na_2_SO_4_, 95.35% for MgSO_4_, 93.83% for CuSO_4_, and 21.66% for NaCl, with permeabilities of 24.45, 22.7, 26.45, and 30.75 L h^−1^ m^−2^ bar^−1^, respectively. Furthermore, the HHST-PDT-TFN membrane exhibited excellent antibacterial properties, proving that the inclusion of a certain amount of NHST uniquely enhances membrane performance and offers exceptional selectivity for mono- and divalent ions, making it highly promising for Li extraction from salt-lake brines.

### 4.4. Surface Grafting Modification of NF Membranes

**Figure 8:**
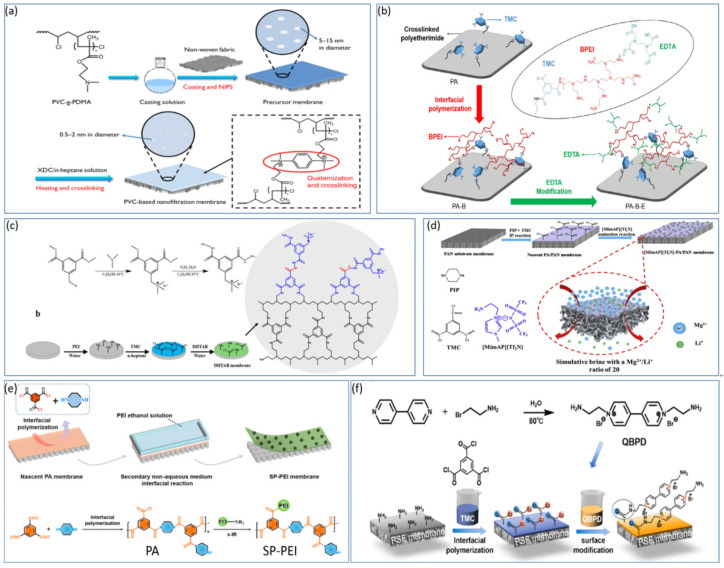
PVC-based NF membrane fabrication process (**a**) [74]; preparation of PA and PA-B membranes via interfacial polymerization (**b**) [75]; synthesis of DHTAB and PEI-TMC membrane fabrication and surface modification (**c**) [76]; [MimAP][Tf2N]-PA/PAN-NF membrane fabrication process and performance in simulated brine (**d**) [77]; synthesis of QBPD and fabrication of the QBPD NF membrane (**e**) [78]; SP-PEI membrane fabrication with surface charge reversal principle and interfacial polymerization of piperazine with trimesoyl chloride (**f**) [79].

Whether involving nanomaterials or metal ion modifications, the high cost and complex synthesis routes limit their widespread and efficient use. To address this challenge, surface grafting modification methods are increasingly attracting attention. Surface grafting involves the use of specific physical and chemical interactions to activate the polymer surface, strategically introducing target functional groups, and thereby enhancing the performance of positively charged NF membranes. In Figure 8a, Fang et al. [74] created a positively charged NF membrane based on PVC grafted with poly(N,N-dimethylaminoethyl methacrylate) (PVC-g-PDMA) through heating and crosslinking treatments The study revealed that the modified NF membrane exhibited a water flux of 9.3 L h^−1^ m^−2^ bar^−1^ and a desalination rate of 93.1% for MgCl_2_ in simulated brine. The quaternization of the membrane surface contributed to its unique pressure resistance and hydrophilicity. Li et al. [75] used crosslinked polyetherimide as a carrier and performed interfacial polymerization with branched polyethyleneimine (BPEI) and TMC to create a positively charged layer (PA-B) composite NF membrane as shown in Figure 8b. The membranes were further grafted with EDTA to increase the selectivity for lithium and magnesium ions. EDTA modification led to significant adsorption of Mg^2+^, with the EDTA-modified NF membrane showing a separation coefficient S (Li/Mg) of approximately 9.2 for simulated brine with a Li^+^/Mg^2+^ ratio of 24 and a membrane flux of 6.0 L h^−1^ m^−2^. The study also demonstrated excellent stability over 36 h in simulated experiments, suggesting good application prospects for salt lakes with high magnesium-to-lithium ratios. Zhu et al. [63] grafted water-soluble PEI onto a polyethersulfone (PES) membrane with tertiary amine groups (PES-TA) and modified it via a green process with polyethylene glycol diglycidyl ether (PEGDGE) crosslinking. The optimal reaction conditions were determined to be 10% PEGDGE and 4% PEI. The results revealed removal rates of 46.2% for NaCl and 94.9% for MgCl_2_, with strong chlorine resistance. Gu et al. [76] modified existing polyamide NF membranes through surface grafting with a multiamine quaternary ammonium salt (3,5-dimethylhydrazine-benzyl trimethyl ammonium bromide, DHTAB) as depicted in Figure 8c. The introduction of DHTAB, with its two symmetric-NH_2_ groups, increased the positive surface charge of the membrane to three times greater than that of the original membrane. The modified membrane could filter 1000 ppm MgCl_2_ under 4 bar pressure with a retention rate of 99.2% and maintained a 94% retention rate for 6000 ppm MgCl_2_. In experiments recovering Li^+^ from a 2000 ppm mixed salt solution of MgCl_2_ and LiCl, the Li^+^/Mg^2+^ separation coefficient reached 60.1. As illustrated in Figure 8d, Wu et al. [77] grafted amino-functionalized ionic liquid 1-(3-aminopropyl)-3-methylimidazoliumbis(trifluoromethanesulfonyl)imide ([MimAP][Tf2N]) onto a polyamide (PA) membrane surface to produce a positively charged NF membrane. Owing to the presence of quaternary ammonium groups from [MimAP][Tf2N], the modified PA membrane was positively charged at pH = 6.4. The membrane exhibited a water permeability of 37.8 L h^−1^ m^−2^, with retention rates of 83.8% for MgCl_2_ and 24.4% for LiCl. After simulated brine with a magnesium to lithium ratio of 20 was processed, the ratio was reduced to 3.5, and the selectivity S (Li, Mg) was 8.12. Feng et al. [78] synthesized a new monomer containing multiple charges (quaternized bipyridine, QBPD) to modify PEI membranes. In Figure 8e, through an amide reaction, the QBPD monomer, featuring dual charge sites and amino groups, was grafted onto the original PEI membrane. The modified QBPD membrane achieved a water flux of 96.6 L h^−1^ m^−2^, which is 2.8 times greater than that of the original PEI membrane, and had a retention rate for MgCl_2_ of 92 ± 3%. Under an operating pressure of 0.6 MPa, the membrane demonstrated high operational stability and good Mg/Li selectivity, reducing the magnesium to lithium ratio from 50 to 8.5 in simulated brine. Lu et al. [79] developed a novel polyamide NF membrane that undergoes a secondary nonaqueous interfacial reaction between the residual -COCl groups on the membrane and PEI, avoiding hydrolysis of the -COCl groups to achieve high amino grafting as shown in Figure 8f. This process effectively reverses the surface charge in situ, creating a selective positive nanolayer on the NF membrane that selectively separates Li^+^ ions from magnesium–lithium mixed solutions. The study also explored the membrane performance after treatment with different molecular weights of PEI and revealed that lower-molecular-weight PEI-treated membranes performed better, with nearly 90% retention of MgCl_2_ and a separation factor S(Li/Mg) > 12, indicating good performance in high magnesium-to-lithium ratio solutions. Vatanpour et al. [80] used a UV-induced method to graft carboxylated polyacrylic acid on the surface of a polyamide NF membrane. This study investigated the impact of different concentrations and reaction times on membrane performance and revealed that the membrane grafted with 50 g/L acrylic acid and irradiated for 5 min under UV light performed best, achieving a membrane flux of 38.8 L h^−1^ m^−2^ and retention rates of 97.43% for Na_2_SO_4_ and 93.4% for NaCl. Peng et al. [81] used a novel bidentate amino electrolyte monomer, brominated diaminated ethyl imidazole (DAIB), to graft and prepare an NF membrane. The results showed that this membrane increased the water flux five times under an operating pressure of 0.6 MPa, achieving a lithium flux of 0.7 mol m^−2^ h^−1^ in simulated brine (MLR = 20) and continuously operating stably for 200 h. Sorekwo et al. [82] used a new tri-quaternary ammonium salt ionic liquid (TQAIL) electrolyte monomer for surface grafting modification to prepare the TQAIL-NF membrane. The results indicated a significant improvement in the separation factor (SF), with the water flux of the modified membrane being four times greater than that of ordinary membranes, reaching 26.11 L h^−1^ m^−2^ bar^−1^. This study improves the separation of lithium and magnesium ions and offers new insights into the use of surface-grafted modified NF membranes for Li extraction from salt-lake brines.

## 5. Anti-Fouling Properties of NF Membranes with High Stability

The stability, lifespan, and efficiency of NF membranes are directly influenced by their flux characteristics. Studies have shown that the performance of NF membranes for Li extraction can be affected by various factors, such as temperature, concentration polarization, organic matter concentration, pH, flow rate and pattern, suspended particles, scale formation, and biological fouling. The contamination of NF membranes can severely reduce their flux, increase the frequency of cleaning, shorten their service life, and severely impact product quality, thus reducing production efficiency. This leads to a decrease in the quality of Li extraction, an increase in impurities, and higher processing costs and complexity, resulting in lower yields. Mitigating fouling in NF membranes has become a focal point of research. NF membranes, being high-pressure membranes, are predominantly affected by surface fouling, which leads to the formation of a filter cake layer. Research indicates that hydrophilic natural organic materials (polysaccharides and proteins) preferentially deposit on the surface of NF membranes to reduce their zeta potential, followed by the deposition of hydrophobic natural organic materials [83]. In the early stages of NF operation, small molecular organic substances can be removed through adsorption, which involves multiple mechanisms, including van der Waals forces, hydrogen bonding, hydrophobic interactions, π-π interactions, and ion-dipole and dipole-dipole interactions [84]. After adsorption saturation, size exclusion becomes the dominant mechanism for NF membranes, accompanied by electrostatic effects to remove charged organic substances and the use of the Donnan effect for selective ion removal. During this process, the small pore size and high retention rate of NF membranes make them highly susceptible to contamination by retained substances, where the accumulation of contaminants can obscure the active layer on the membrane surface [85].

**Figure 9:**
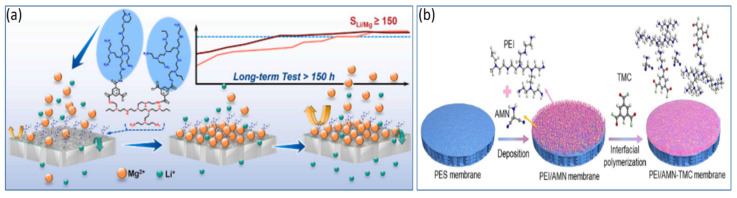
Separation mechanism and stability of PEI-70000/PA/PES membrane and its separation factor (**a**) [88]; schematic diagram of PEI/AMN-TMC membrane fabrication (**b**) [89].

Zhu et al. [86] investigated the effectiveness of composite NF membranes in removing organic micropollutants via two different types of membranes. They reported that the NF270 membrane exhibited higher permeability compared to NF90. Additionally, the performance of the NF90 membrane was less affected by contaminants, and it showed weaker removal of organic micropollutants than the NF270 membrane. The NF90 membrane demonstrated higher levels of fouling resistance. Structurally, spiral-wound NF membranes have a relatively high packing density, whereas hollow-fiber NF membranes have a relatively dense and smooth surface layer, which reduces the likelihood of fouling and effectively enhances the membrane’s fouling resistance [87]. Furthermore, the hydrophilicity of the membrane surface plays a crucial role in membrane fouling. Through electrostatic attraction and hydrogen bonding, the hydrophilicity of the NF membrane surfaces continues to increase, facilitating the formation of a hydration layer. To date, there are still diverse opinions on the theory of membrane surface hydrophobicity, but it is generally believed that most NF membranes are hydrophobic, and efforts to increase the surface hydrophilicity of NF membranes are beneficial for extending their lifespan and anti-fouling capabilities. The synergistic effects of graphene oxide and vanillin in improving membrane permeability and antifouling properties are gaining increasing attention. Zhao et al. [88] fabricated a PEI-70000/PA/PESNF membrane via interfacial polymerization via PEI and TMC and optimized the pore structure and positive charge density of the active layer by varying the molecular weight of PEI and the surface amine grafting density. This achieved the selective separation of Mg^2+^ and Li^+^ as shown in Figure 9a. The study revealed that the membrane could operate continuously and stably for 150 h at feed concentrations of 2000 or 3000 ppm, maintaining a permeate flux of 20 L·m^−2^·h^−1^·MPa^−1^ and reducing the Mg^2+^/Li^+^ ratio to 0.32, demonstrating the potential for Li extraction from salt-lake brines with high Mg^2+^/Li^+^ ratios and exhibiting good long-term stability. Li et al. [89] explored the addition of amino nitriles (AMNs) between PEI chains to modify the polyamide layer and fabricate a PEI/AMN-TMC membrane as depicted in Figure 9b. The -NH_2_ groups present in AMN molecules can be protonated to −NH^3+^ in aqueous solutions, increasing the positive charge of the membrane and loosening its structure to increase water permeability. This study also used density functional theory (DFT) methods to predict and research the affinity of AMN and PEI molecules for water and their repulsion towards Li^+^ and Mg^2+^. The resulting PEI/AMN-TMC membrane exhibited exceptional water permeability, 2.9 times greater than that of traditional membranes, at 13.1 L·m^−2^⋅h^−1^⋅bar^−1,^ and a high MgCl_2_ retention rate of 97.53%, effectively reducing the high Mg^2+^/Li^+^ ratio from 20–0.8, with an S(Mg^2+^/Li^+^) value of 26.7. The membrane displayed up to 7 days of stable separation performance, as well as antiscaling and antibacterial properties during the experiments. This material demonstrated excellent performance and stability for lithium recovery from salt-lake brines, showing strong potential for application.

## 6. Conclusions and Prospects

As global energy transitions accelerate and the demand for lithium surges, salt-lake brine has emerged as the dominant resource for sustainable lithium recovery. NF technology, with its energy-efficient, environmentally friendly, and scalable characteristics, has become a cornerstone for selective Li^+^ extraction from brines with high Mg^2^^+^/Li^+^ ratios. Recent advancements in cationic NF membranes enabled by surface functionalization, nanomaterial incorporation, and copolymer engineering have significantly improved the Mg^2^^+^/Li^+^ separation performance, leveraging size exclusion, Donnan effects, and dielectric repulsion mechanisms. Notable progress has been made in the development of amine-functionalized polyamide membranes, metal ion-doped nanocomposites, and graphene oxide hybrid structures, which demonstrate promising potential for industrial applications.

However, several critical challenges hinder large-scale commercialization: (1) membrane stability: long-term operational durability under harsh brine conditions remains inadequate, necessitating advancements in fouling-resistant coatings and chemical stability; (2) cost-effectiveness: complex fabrication processes and expensive raw materials (e.g., amine monomers) limit economic viability; scalable manufacturing techniques are urgently needed; (3) system integration: current NF systems lack modularity and process intensification, requiring innovative hybrid configurations (e.g., NF–adsorption–electrodialysis) to optimize energy efficiency; and (4) selectivity enhancement: while Mg^2^^+^/Li^+^ separation factors have improved, achieving >99% Li^+^ purity remains challenging for hypersaline brines.

Advancing lithium recovery from salt-lake brines requires a multifaceted approach, starting with the development of next-generation membranes incorporating ion-imprinted polymers and ion-conductive frameworks that selectively facilitate Li^+^ transport while minimizing Mg^2^^+^ interference through tailored pore chemistry. Concurrently, process optimization strategies leveraging AI-driven modeling tools will enable the design of energy-efficient, self-cleaning NF systems with real-time fouling monitoring and adaptive operational capabilities. Sustainable material innovation should focus on biobased polymers (e.g., chitosan-grafted polyamides) and recyclable nanocomposites to reduce the environmental footprint without compromising separation performance. Moreover, techno-economic analysis through lifecycle assessments will be critical for balancing improvements in membrane performance with cost reductions, ensuring scalability and alignment with industrial requirements. Collectively, addressing these challenges will solidify NF technology as the cornerstone for sustainable lithium extraction from complex brines while driving transformative breakthroughs in polymer science.

## Figures and Tables

**Table 1:** Lithium content of typical salt lakes in the world.

Lake Name	Region/ Country	Lake Type	Lithium Content	References
1. Great Salt Lake	Utah, USA	Chloride type	Mean 48 mg/L, Resource 0.44 Mt Li	[3]
2. Clayton Valley	Nevada, USA	Sulfate type	Mean 146 mg/L, Resource 0.05 Mt Li	[4]
3. Salar de Uyuni	Potosi, Bolivia	Chloride type	Mean 715 mg/L, Resource 9.00 Mt Li	[5]
4. Salar de Atacama	Antofagasta, Chile	Sulfate type	Mean 1880 mg/L, Resource 9.60 Mt Li	[6]
5. Salar de Uyuni	Salar de Uyuni, Bolivia	Sulfate type	Mean 715 mg/L, Resource 9.00 Mt Li	[5]
6. Zhabuye Salt Lake	Shigatse, Tibet, China	Carbonate type	Mean 1467 mg/L, Resource 1.53 Mt Li	[7]
7. West/East Taijinar Lake	Qinghai, China	Magnesium sulfate subtype	Mean 171 mg/L, Resource 0.90 Mt Li	[7]
8. Qarhan	Qinghai, China	Chloride type	Mean 126 mg/L, Resource 4.82 Mt Li	[8]
9. Salar del Hombre Muerto	Catamarca, Argentina	Sulfate type	Mean 628 mg/L, Resource 3.61 Mt Li	[5]
10. Salar de Coipasa	Potosi, Bolivia	Sulfate type	Mean 258 mg/L, Resource 0.20 Mt Li	[5]

**Table 2:** Lithium content of typical lithium mines in the world.

Salt Mine Name	Region/Country	Deposit Type	Lithium Content	References
1. Manono	Tanganyika, DR Congo	Pegmatite	Average 7563 ppm, Resource 3.78 Mt Li	[9]
2. Cyprees—Zeus	Nevada, USA	Volcano-sedimentary	Average 1189 ppm, Resource 0.48 Mt Li	[10]
3. Greenbushes	Western Australia	Pegmatite	Average 6960 ppm, Resource 2.84 Mt Li	[11]
4. Earl Grey—Mt Holland	Western Australia	Pegmatite	Average 7162 ppm, Resource 1.23 Mt Li	[12]
5. Mina de Cachoeira	Minas Gerais, Brazil	Pegmatite	Average 6496 ppm, Resource 0.72 Mt Li	[13]
6. Bougouni	Sikaso, Mali	Pegmatite	Average 5243 ppm, Resource 0.06 Mt Li	[14]
7. Mt Cattlin	Great Southern Australia	Pegmatite	Average 6044 ppm, Resource 0.07 Mt Li	[15]
8. Manna	Western Australia	Pegmatite	Average 5197 ppm, Resource 0.10 Mt Li	[16]
9. Rhyolite Ridge	Nevada, USA	Volcano-sedimentary	Average 1600 ppm, Resource 0.12 Mt Li	[17]
10. Jadar	Western Serbia	Volcano-sedimentary	Average 7795 ppm, Resource 0.67 Mt Li	[18]

## Data Availability

No new data were created or analyzed in this study.
